# Marie Poussepin’s Influence on Nursing from Her Vocation of Service and Charity*

**DOI:** 10.17533/udea.iee.v41n2e06

**Published:** 2023-08-18

**Authors:** Paula Andrea Duque, Sandra Milena Campiño, Mildred Guarnizo-Tole, Daniela Escobar-Peláez

**Affiliations:** 1 Nurse, Master’s. Email: paduque@ucm.edu.co. Universidad Católica de Manizales. Manizales -Caldas (Colombia). https://orcid.org/0000-0001-7237-6195 Universidad Católica de Manizales Universidad Católica de Manizales Manizales Caldas Colombia paduque@ucm.edu.co; 2 Nurse, Master’s. Email: scampino@ucm.edu.co. Universidad Católica de Manizales. Manizales -Caldas (Colombia). https://orcid.org/0000-0002-8754-4777 Universidad Católica de Manizales Universidad Católica de Manizales Manizales Caldas Colombia scampino@ucm.edu.co; 3 Nurse, PhD. Universidad el Bosque. Bogotá, Colombia. E-mail. guarnizomildred@unbosque.edu.co https://orcid.org/0000-0002-2112-311X Universidad El Bosque Universidad el Bosque Bogotá Colombia guarnizomildred@unbosque.edu.co; 4 Nurse. E-mail: daniela.escobar1@ucm.edu.co Universidad Católica de Manizales. Manizales -Caldas (Colombia). https://orcid.org/0009-0008-1991-3156 Universidad Católica de Manizales Universidad Católica de Manizales Manizales Caldas Colombia daniela.escobar1@ucm.edu.co

**Keywords:** nursing care, nuns, history of nursing, hermeneutics, atención de enfermería, monjas, historia de la enfermería, hermenéutica, cuidados de enfermagem, freiras, história da enfermagem, hermenêutica

## Abstract

**Objective:**

This work sought to describe the influence of Marie Poussepin on Nursing from her vocation of service and charity.

**Method:**

Historical-hermeneutic study with participation by 15 Dominican Sisters of Charity in the Presentation of the Blessed Virgin from the city of Manizales and Bogotá, Colombia, who answered semi-structured interviews. The information was gathered and recontextualized via the open and axial coding system through ATLAS.ti9 software. During the interpretation procedure, copying, intensive reading, note taking, analysis, first epigraph of the report, coding, grouping, and determination of categories was made, conducting information triangulation with existing evidence.

**Results:**

Three categories emerged*: Responding to the call of Jesus through service to the community*; *Under the legacy of charity, respect for life and the dignity of human beings*, and *Caring for life as a foundation of nursing*. The second category formulated the description that integrates the course of life and objectives of the institutions where the nuns interviewed work.

**Conclusion:**

The legacy by Marie Poussepin to nursing care has been manifested since the foundation of the work, influencing the disciplinary work through the vocational commitment of those who make up the congregation, imprinting a character of service and respect for others, in response to the love of God.

## Resumen

**Objetivo:**

Describir la influencia de Marie Poussepin para la enfermería, desde su vocación de servicio y caridad.

**Método:**

Estudio histórico-hermenéutico. Participaron quince Hermanas de la Caridad Dominicas de la Presentación de la Santísima Virgen de la ciudad de Manizales y Bogotá, Colombia, quienes respondieron a entrevistas semiestructuradas. Se recogió y recontextualizó la información mediante el sistema de codificación abierta y axial del software ATLAS.ti9. Durante el procedimiento de interpretación se hizo copia, lectura intensiva, toma de notas, análisis, primer epígrafe del informe, codificación, agrupación y determinación de categorías, y se llevó a cabo la triangulación de la información con la evidencia existente.

**Resultados:**

Emergieron tres categorías*: Dando respuesta al llamado de Jesús mediante el servicio a la comunidad*; *Bajo el legado de caridad, respeto por la vida y la dignidad del ser humano* y *Cuidado de la vida como fundamento de enfermería*. En la segunda categoría se realizó la descripción que integra el curso de vida y objetivo de las instituciones donde laboran las religiosas entrevistadas.

**Conclusión:**

El legado de Marie Poussepin, al cuidado enfermero, se ha manifestado desde la fundación de la obra y ha influido en la labor disciplinar mediante el compromiso vocacional de quienes integran la congregación, e imprime un carácter de servicio y respeto por el otro en respuesta al amor a Dios.

## Resumo

**Objetivo:**

Descrever a influência de Marie Poussepin para a enfermagem, a partir de sua vocação de serviço e caridade.

**Método:**

Estudo histórico-hermenêutico. Participaram quinze irmãs da Caridade Dominicanas da Apresentação da Santíssima Virgem da cidade de Manizales e Bogotá, Colômbia, respondendo a entrevistas semiestruturadas. As informações foram coletadas e recontextualizadas por meio do sistema de codificação aberta e axial por meio do software ATLAS.ti9. Durante o procedimento de interpretação, foram feitas cópia, leitura intensiva, anotações, análise, primeira epígrafe do relato, codificação, agrupamento e determinação de categorias, realizando-se a triangulação das informações com as evidências existentes.

**Resultados:**

Emergiram três categorias*: Respondendo ao chamado de Jesus através do serviço à comunidade*; *Sob o legado da caridade, o respeito à vida e à dignidade da pessoa humana* e *o cuidado à vida como fundamento da enfermagem*. Na segunda categoria, foi feita a descrição que integra o percurso de vida e objetivo das instituições onde trabalham as freiras entrevistadas.

**Conclusão:**

O legado de Marie Poussepin para o cuidado de enfermagem se manifesta desde a fundação da obra, influenciando o trabalho disciplinar através do compromisso vocacional daqueles que compõem a congregação, imprimindo um caráter de serviço e respeito ao próximo, em resposta ao amor a Deus.

## Introduction

From everyday female environments, where the domestic tasks prevail, caring for the sick and the helpless has been conducted, in which women intuitively used elements, like water, vapor, fire, animal skins, and medicinal plants to satisfactorily perform their work.^1^^,^^2^ However, history has granted men leading roles concerning health care and, therefore, made it easier for their work to be paid and hold social recognition.^2^ It is known that the first Nursing school began in India 250 years B.C., and only men were sufficiently pure to become nurses. In Athens, around 300 B.C, law banned women from engaging in work related to medicine and obstetrics; therefore, such work belonged to the male gender based on the religious and military spheres.^3^ Nevertheless, despite the prohibitions of the times, it has been documented that women dedicated themselves to caring for the sick who had no financial resources, thus, their remuneration was given with food or lodging.^1^

During the Roman Empire, "the Parabalani" were consolidated; these were men belonging to the Christian communities who founded hospitals and cared for the sick.^3^ In Christianism, women assume the role in caring for the ill, bringing the exercise of charity to the neediest. For this reason, these communities were pioneers of "nosocomiums", or places where care was offered without distinction of creed, nation or socioeconomic condition. Due to the foregoing, during the Middle Ages, the role of women in care and attention to the sick and disabled was recognized.^4^

In 1952, Peplau established the first conceptual nursing model denominated “Interpersonal Relations in Nursing”, which describes the nurse-patient relationship as a means to promote therapeutic interpersonal development, with the behavior and emotions of the professional being relevant to care for the patient until the resolution of the disease is achieved.^5^ It designates that, to care, vocation, service and charity are required. Colliére^6^ describes four stages of care, starting from domestic, vocational, technical, and professional care; each giving way to the next as a result of the needs, challenges, and evolutions (intellectual, scientific, and social) of each time. 

Within this context, by the mid-17^th^ century in Dourdan France, Marie Poussepin was born, founder of the Congregation "Dominican Sisters of Charity of the Presentation of the Blessed Virgin".^7^ During her childhood, Poussepin glimpsed the Christian foundations of her project, thanks to her rapprochement with the Daughters of Charity and Ladies of Charity of Saint Vincent de Paul, so from her position as Treasurer and Superior of the Confraternity she decided to dedicate herself completely to charity.^8^ Later, she founded a community based on Christian principles and regulations, where girls were educated and taught to work according to their age; a circumstance that raised awareness of the importance of work. These beneficiaries became the first teachers, nurses and Sisters of the Congregation. With respect to caring for the sick, Poussepin mainly offered her care to the poor. In Sainville, prior to 1698, there were no hospitals, so nuns went to rural areas that needed care interventions or medications. She also created a pharmacy, which provided home remedies based on medicinal herbs, performed cures, and designated nursing care according to the prevailing need.^8^

With Louis XIV’s mandate, hospital conformation was regulated; therein, Poussepin instructed the women who were part of the religious community in the exercise of charity and care of the sick, describing that disease should be treated according to each person’s social and cultural patterns, values, and beliefs. Additionally, she urged the application of knowledge, skills, and attitudes to dedicate oneself with passion, justice, quality, and intelligence in care.^9^ According with the aforementioned, it is valid to relate the domestic and vocational care of nursing with the work by Poussepin, given that for over 300 years it has been impacting the lives of thousands of women with a vocational, selfless, and charitable sense for humanity. Her global influence has promoted the conformation of organizations extended in four continents, which have helped the lives of children, youth, adults, and the elderly who live in precarious and disabled environments. Given this, the present study sought to describe the influence by Marie Poussepin on Nursing, taking as a starting point the perspective of life stories of the Dominican Sisters of Charity of the Presentation of the Blessed Virgin. 

## Methods

*Type of study and sample.* Historical-hermeneutical study, conducted in 2022 in Colombia. Oral histories were used as a research strategy to gather information from the Dominican Sisters of Charity of the Presentation of the Blessed Virgin to reconstruct their experiences, anecdotes, customs, and understand the influence by Poussepin on current nursing. To choose the participants, a convenience sampling was used to select the informant nuns and their different roles within the community, following the criterion of intersubjectivity to guarantee the representativeness of the testimonies. As inclusion criteria for the participants, it was established that they should have five or more years as members of the community and function as nurses or educators in the different trajectories during the course of their lives. In all, 15 nuns participated, there were no refusals or withdrawal from the study. 

*Ethical aspects.* In compliance with the ethical requirements, endorsement from the Research Ethics Committee at Universidad Católica de Manizales was obtained, through Agreement 179. The participants were contacted in clinics in Manizales and Bogotá, Colombia, in social works and in a kindergarten in the city of Manizales. All participants signed the informed consent after being explained the purpose of the study and manner of execution and participation. They were told that there were no good or bad answers, and that all information collected would be confidential - hence, each interview was identified with an alphanumeric code and data alluding to the place, the institution and the informants were omitted - participation was voluntary. It must be clarified that neither researchers nor participants had any type of relationship before the execution of this meeting; therefore, there was no conflict of interest. 

*Data collection technique.* To obtain the reports, semi-structured interview was used after an appointment was set up via telephone call. The interviewers were two research-teaching nurses with PhD degrees in Health Sciences and Pedagogy; the third held an MSC degree in Education. The three are recognized as researchers by COLCIENCIAS and, besides being the principal researchers, they also designed and executed the study protocol. The participating nuns were approached individually with nine descriptive-type questions during a single occasion, no type of recording was made at the request of the participants; the interviewing researcher took notes of what the participants said during and at the end of each meeting. Efforts were made to maintain a warm and trusting atmosphere. The average duration of the interviews was approximately 30 minutes. The notes were read aloud after the interview so that the informants could make the corrections deemed pertinent. 

*Information analysis plan.* After transcribing the interviews, the three researchers began the analysis of the stories. Information was collected and recontextualized through the open and axial coding system using ATLAS.ti9 software. During the interpretation procedure, copying, intensive reading, note taking, analysis, first epigraph of the report, coding, grouping, and determination of categories were made towards analyzing the symbolic efficacy of the stories, triangulating the information with existing evidence, so that the results were contrasted with similar or divergent positions. 

## Results

The work obtained 15 interviews from nuns belonging to the congregation of Dominican Sisters of Charity of the Presentation of the Blessed Virgin, ranging between 30 and 93 years of age. The participant with the most experience in congregational life had around 60 years and the one with the least experience only had 5 years. Of all the nuns, five were nurses, five were teachers, and the remaining five supported care tasks of the Congregation from their area of expertise. 

**Table 1:** Demographic data of the participants

Number	Age	Years in the congregation	Profession	Institution where they work or worked
1	53	35	Pedagogist	University
2	46	28	Nursing aide	Clinic
3	52	36	Bachelor of Social Sciences	School
4	34	9	Nurse	Clinic
5	30	5	Attorney	School
6	87	60	Bachelor of Social Sciences	School
7	74	46	Nurse	Clinic
8	35	16	Nurse	University
9	78	39	Public Accountant	University
10	93	59	Nurse	School
11	89	60	Nursing aide	Clinic
12	51	34	Nursing aide	Clinic
13	76	38	Pedagogist	Kindergarten
14	39	20	Pedagogist	Kindergarten
15	48	27	Nurse	Clinic

During the analysis process, three categories emerged, grouped as follows: I. Responding to the call of Jesus through service to the community, 2. Under the legacy of charity, respect for life and the dignity of human beings, and III. Caring for life as a foundation of nursing. Each of these is described ahead. 

### Category 1. Responding to the call of Jesus through service to the community

This category exposes that the heiresses of Poussepin's legacy recognize in their work the connotation of their religiosity, given that they integrate into their congregational act the function of care during a person’s illness. Within these actions, it is possible to identify the search for bodily and spiritual healing: *I think that Marie's passion for service to life is impressive, she rescued the particular simplicity she has towards caring for the sick... from her vision of faith, she found the presence of Jesus and that is why she served the sick* (PE1); *She was a prosperous woman, interested in the people devastated by war and the misery in Saint Ville; in her time, she dropped everything to help. She made me question myself, and I began to have those feelings of wanting to be just like her* (PE 4).

Also, the legacy given is aimed at the contributions made to the life project of the nuns, and how this has guaranteed the work of service from the different areas where they operate within their group: *She left me charity and love for Jesus as a legacy; I am here because I have love to serve our Lord through service to others* (PE 2); *I joined the Congregation because I heard the call of God. The Christian formation in my family and the testimony of the nuns greatly influenced on the desire to serve* (PE 10); *I discovered that on a vocational level, the charisma of our foundress is the second greatest gift that God gave me* (PE6).

Under this philosophy, care work is perceived as integral because it not only calms bodily pain. Interventions take into account different human needs. In addition, care work is not always given from the degree, because the service spirit is essential to achieve success: *Many of us are not nurses, but we have soothed the souls of children, collaborators; you are not a nurse just to respond to a physical health situation, but to take care of a person’s soul, and that is what we do* (PE7); *The truth is that Marie taught us something that we all must have and we have seen it since the time of Jesus: being moved by compassion and having a fervent charity for people* (PE11). 

### Category 2. Under a legacy of charity, respect for life and the dignity of human beings

This category recognizes that care work frame-worked within Poussepin’s legacy is not only limited to providing bodily or spiritual needs. This panorama dignifies human life during illness and health. On the other hand, in the different scenarios where the nuns carry out their work, life and its respect are the center of the entire work. 

Community social work: *We conduct guidance through home visits. I recruit pregnant women, the elderly or children and refer them to foundations where they can be provided help* (PE8); *We support morally and spiritually the people who come to us* (PE8). The presence of the nuns in the in the works linked to care and education, it can be seen in the works that they lead and whose beneficiaries are at different moments of their life course. 

Kindergarten: *We live Marie's spirituality, we transmit to children the tenderness and love our mother had for them* (PE7); *Next to health care, schools were formed. Marie taught the girls to read and to care for others* (PE12).

Clinical care: *Marie taught that it is about being concerned for the care of life and a deep sensitivity towards the vulnerable state of human beings in their moment of illness.* (PE4); *She highlighted from nursing not only the technique, but also that of providing careful treatment to patients* (PE 13). 

Educational community: *The Nursing Program is the essence of the gospel (PE10); For Marie, everything became a possibility to heal the body, the heart, the spirit, the soul. She tells the nuns who are nurses that sometimes more good is done by healing the spirit because of that association between illness and interiority* (PE5); *The entire strategic platform of the clinic is based on Marie's charisma: vision, mission, and corporate values* (PE10); *At university, everything is related to the word care, whenever we get close to a person, we consider them sacred ground* (PE5). The contributions referred to indicate the close relationship among religious work, care, and education. These assignments are representative and widely recognized inside and outside the Congregation. 

### Category 3. Caring for life as a foundation of nursing

For this category, care is recognized as the essence of nursing and, at the same time, it is typified as the extension of Poussepin's missionary work through a series of links established in the moments of care practice. The institutions in which the participants work are places of service to others in different ways: *The health care worker is a continuation of Marie Poussepin’s charisma* (PE12); *The exercise of health in an institution is a ministry within the church. We make an announcement to our patients: through humane treatment and warm service* (PE12); *The strategy we have is the interpersonal relationship... that relationship born deep within our being because it is God's gift to each one and He himself helps us to put it into practice* (PE13). 

## Discussion

Marie Poussepin’s legacy, given through the activities carried out by the Congregation, has deepened the foundations in the academic and practical aspects of nursing, as well as in the moral, spiritual, and human foundations necessary to execute nursing care in today's society, strengthening the technical and professional era of a practice that emerged empirically. It should be noted that the congregation of the Dominican Sisters of Charity of the Presentation of the Blessed Virgin constitutes an organization that promotes the active life of its nuns, favoring their spiritual development in the light of the Gospel and opting for their academic and professional enrichment. Hence, within the organization, there are three fields of action related to health, education, social, and parish ministry that are consistent with the life course approach defined by the Ministry of Health as "an approach that addresses moments of life, recognizing that human development and health outcomes depend on the interaction of cumulative experiences and present situations of the individual influenced by the family, social, economic, environmental, and cultural contexts.^10^

The findings coincide with a study on anthropology and care, which explained how the development of the culture of care was not only carried out through Christianity, but through all religions that showed concern for the sick. In this aspect, it is worth mentioning that the Christian culture of care addressed the idea of hospitality.^11^ It should be stated that, regarding the notion of hospitality established by nursing over centuries, not only the technical functions of its work were addressed, but also the skills for the exercise of charity, considering that care should be exercised by consecrated individuals.^7^ In other words, and bearing in mind the current practical and moral development of the profession, comprehensive care arises when a genuine relationship is established between the caregiver and the person being cared for. Hence, Christian charity is based on the execution of actions, passing the care of the poor and sick to an important social level, that is, what was once an occupation of slaves became a sacred vocation. Service, through caring for the neediest, went on to be seen as a duty of men and women who profess a faith.

History recounts Marie Poussepin’s childhood around the wars and plagues that left the population in precarious conditions of job insecurity, health, and economy. Poussepin, thanks to her mother and her closeness with the gospel, set out to serve the sick from a very young age, learning to visit them and provide them with food.^8^ The foregoing is similar to the foundation upon which the participants have based their work within the congregation, where the presence of these workers is representative, not only in the domestic scenario of the needy, but in those scientifically endorsed, such as they are: the hospital, the school, and the university.

Nursing participates actively in caring for patients, and when it is aimed at strengthening the family environment, it improves people's self-esteem and level of confidence, being able to potentiate their possibilities and the positive consequences.^12^ This condition is consistent with the work by the participants in contexts unrelated to illness, and where it is possible to generate greater social responsibility. In this regard, a study exposes the emotional support provided by the nursing staff to hospitalized patients as relief to the soul. Reiterating that nurses require a high level of sensitivity to interpret the patient’s verbal and non-verbal manifestations associated with their perception regarding the illness, the hospitalization and treatment process, and refer that emotional support should be understood as a form of careful.^13^

Moreover, Fernandes and Siles ^11^ consider that female social work arose as a response to social emergencies, which at the time produced the fulfillment of care for free or "volunteer work". Said antecedent impregnated it in his course, mainly under the leadership of the church, as an unpaid activity carried out by those who perceived a group of people in deprived situations, who demanded charity.^14^ This evidence is viable and latent in the discourse used by the participants, given that they support their function and philosophy on this vocational principle that seeks to overcome discomfort or vulnerability. For this reason, the Congregation is the non-profit group in charge, in the city of Manizales, of visiting areas and attracting individuals in vulnerable conditions (women heads of household, people with disabilities, children or the elderly in social abandonment), to refer them to institutions that have the pertinent economic and social conditions to favor their integral state of health. In its social works, it promotes continuity in health care to the community through the dispensary, which provides medications under a medical formula to people who do not have the resources to obtain them. 

Likewise, it is highlighted that the labor of care in the diverse settings of missionary work is not disassociated from health regulations. On the contrary, the function of the Congregation is viable because it recognizes the family as subjects of care and health care as a permanent process, focused on disease prevention. Such an act permits providing optimal tools for each person from their community to manage their self-care,^15^ thereby, it is worth highlighting this organization for its contribution in promoting health and strengthening self-care in the community. That is, the missionary work, provided around care, recognizes that health problems are generated or enhanced by multiple factors that must be intervened from childhood, to promote adequate development through optimal educational conditions that offer strategies for the correct physiological, mental, emotional, and spiritual development of people. This discovery is true compared to the educational work that has historically been conducted and that has been linked to health care. 

Another fact found and of interest is that the missionary work around care has sought, over the years, to recognize that health care must be comprehensive and not fragmented. Authors, like Castro *et al*.,^16^ refer to the vitality of committing and getting involved in the process and never leaving aside the humanization of the task in favor of improving health results. Thus, the data analysis of the present study is aligned with that described in the literature, given that through reports by the participants, caring becomes visible when someone's life becomes important for the other, and they decide to participate in it. It is through acts that result from understanding of the state of others and the purpose of helping them.^17^

Although the humanization of health services presumes being an elemental component during the individual's health restoration process, Castillejos *et al.*,^18^ express that the relationship of emotional intelligence with the care provided by nurses contributes to the emotional, spiritual, and aid components. Added to the fact that it is a determinant to improve the quality of care for people hospitalized. Then, the work by Poussepin corresponds to the emerging trends in human healthcare, despite adversities present during this commendable work and which indicate the workload, imposition of administrative activities that prioritize indicators, costs and protocols, development of individualistic thinking, inconsistency between theory and practice, and lack of recognition are barriers that have made us think that values and principles, among others,^19^^,^^20^ have deteriorated care, mainly institutionalized care. Consequently, vocation, will, patience, respect for the dignity of others, charisma, warmth and compassion are requested from the environment where the study phenomenon was addressed.^21^

Nursing epistemology encompasses the comprehensive care of humans through health promotion, disease prevention, and timely diagnosis and treatment in the required dimension. In response to the aforementioned, the individual's physiological, spiritual, and emotional well-being is favored if the vulnerability of life is considered when the required care is not received.^22^

This study concludes that Marie Poussepin's contribution to nursing care has been experienced since the foundation of the work. It is evident that said work is not clearly limited to the institutionalization of care; on the contrary, care is present in various contexts where nuns execute their mission. In this sense, the legacy in mention has influenced nursing from the religiosity, responsibility, and vocation of those comprising this congregation, imprinting a character of service and respect for others, in response to the love of God. Historically, it has not been limited to a degree in the area, given that, from the very formation in the community, women have been taught and based on care; it is then a professional act and responsibility towards others that deserves an exhaustive practice. 

The limitation in this study lies in that not all the participants were nurses; nonetheless, under the religious legacy, the act of caring is a primordial part of their work.
